# The impact of sex and gender on immunotherapy outcomes

**DOI:** 10.1186/s13293-020-00301-y

**Published:** 2020-05-04

**Authors:** Sabra L. Klein, Rosemary Morgan

**Affiliations:** 1 W. Harry Feinstone Department of Molecular Microbiology and Immunology, The Johns Hopkins Bloomberg School of Public Health, Baltimore, MD USA; 2 Department of International Health, The Johns Hopkins Bloomberg School of Public Health, Baltimore, MD USA

**Keywords:** Autoimmunity, Cancer, Checkpoint therapy, CTLA-4, Influenza vaccine, Rheumatoid arthritis, PD-1/PD-L1, Tumor necrosis factor (TNF) inhibitor

## Abstract

Immunotherapies are often used for the treatment, remission, and possible cure of autoimmune diseases, infectious diseases, and cancers. Empirical evidence illustrates that females and males differ in outcomes following the use of biologics for the treatment of autoimmune diseases, e.g., rheumatoid arthritis (RA), infectious diseases, e.g., influenza, and solid tumor cancers. Females tend to experience more adverse reactions than males following the use of a class of biologics referred to as immunotherapies. For immunotherapies aimed at stimulating an immune response, e.g., influenza vaccines, females develop greater responses and may experience greater efficacy than males. In contrast, for immunotherapies that repress an immune response, e.g., tumor necrosis factor (TNF) inhibitors for RA or checkpoint inhibitors for melanoma, the efficacy is reportedly greater for males than females. Despite these differences, discrepancies in reporting differences between females and males exist, with females have been historically excluded from biomedical and clinical studies. There is a critical need for research that addresses the biological (i.e., sex) as well as sociocultural (i.e., gender) causes of male-female disparities in immunotherapy responses, toxicities, and outcomes. One-size-fits-all approaches to immunotherapies will not work, and sex/gender may contribute to variable treatment success, including adherence, in clinical settings.

In medical research, there is a long history of not analyzing or reporting differences between females and males in the presentation and progression of disease as well as in the prophylactic or therapeutic treatment of disease. This occurs, despite growing evidence that sex differences exist in the biochemistry and functioning of every organ system in the body [1]. Male-female differences also exist in the presentation and prognosis of diverse diseases ranging from Alzheimer’s disease and multiple sclerosis to cardiovascular disease and asthma, to name a few [2]. The underappreciation of how biological and even social/cultural differences between males/men and females/women (males and females denote sex and are the preferred terms for the remainder of this paper unless specifically referring to gender) can serve as disease treatment modifiers (Fig. 1) is a direct reflection of the history of excluding females from biomedical and clinical studies—a practice that began in 1977 when the U.S. Food and Drug Administration (FDA) published guidelines advising that females of childbearing potential be excluded from drug trials [3]. One goal was to protect pregnant females and their fetuses from adverse outcomes, but the unintended consequence was complete exclusion of females despite advocacy around informed consent, including autonomy to make independent decisions about (1) trial participation, (2) risks and benefits to the fetus, (3) medical understanding of sex differences, and (4) societal need to understand how drugs work in the larger population, which includes females [4]. These recommendations resulted in inadequate representation of females in clinical trials for decades. Thus, sex and gender have effects on broad research structures and processes, including what gets researched, what gets funded, and what or who is ultimately excluded. The historical exclusion of females from drug trials and ignoring of sex differences is evidence of how gender bias present within research structures and processes that can have a negative effect on women’s health.

**Fig. 1:**
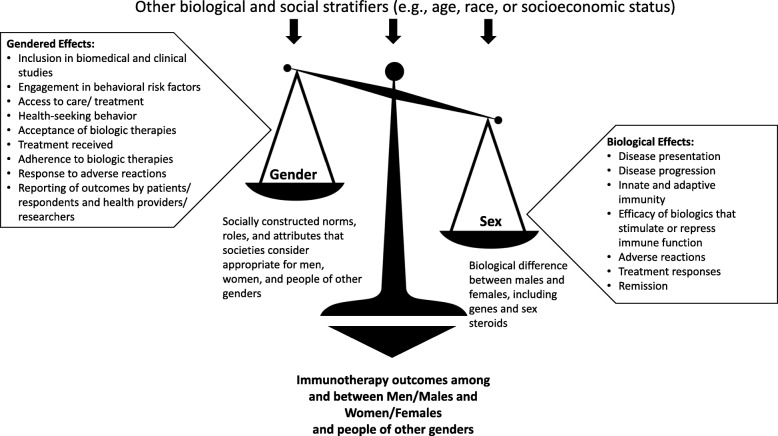
Both sex- and gender-based factors contribute to differences between females/women and males/men and should be considered in biomedical research**.** Gender influences such things as differential inclusion of individuals in biomedical and clinical studies, engagement in behavioral risk factors, access to care and treatment, health-seeking behaviors, and acceptance of immunotherapies, such as vaccines, treatment received, adherence to therapies, response to adverse reactions, and the reporting of outcomes by both patients and health providers and respondents and researchers. Biological sex can impact the pathogenesis of the targeted diseases and immune responses to immunotherapies as well as development of adverse reactions. These outcomes will be influenced by the ways in which gender and sex intersect with other biological and social stratifiers such as age, race, disability, socioeconomic status, and other factors. Together, both sex and gender, and their intersection with other biological and social stratifiers, contribute to differential efficacy of immunotherapies

In the early 1990s, the FDA and the National Institutes of Health (NIH) in the USA, with advocacy from U.S. Congresswomen, recommended that clinical trials include female subjects [4, 5]. Although females are now included in clinical trials of drugs, devices, and biologics, there remains inadequate analysis of whether outcomes differ between females and males [6]. In addition, there is little to no disaggregation of data by sex and other social or biological stratifiers, such as race and age (Fig. 1).

Of drugs withdrawn from the U.S. market from 1997 to 2000, the U.S. Government Accountability Office (GAO) reported that 8 out of 10 drugs taken off the market had greater adverse effects in females [7]. In 2015, the U.S. GAO documented that while more females than males currently enroll in NIH-funded clinical research, the NIH does not ensure that these studies are designed to identify differences between males and females in disease processes and responses to treatment. Preclinical studies in animal models and cell culture systems could help to prevent these costly mistakes, but here too, analysis of potential sex effects has been lacking. In the USA, the NIH implemented a policy in 2016 that requires investigators seeking federal funds for preclinical research to consider how biologic sex impacts research findings to enhance rigor and reproducibility of results.

The ultimate goal for changing policies about inclusion of females in biomedical, clinical, and public health research is to determine whether disease presentation, progression, and treatment are different for females than males and then address the mechanisms mediating these differences. Despite the simplicity of this goal, in the biomedical sciences, sex/gender-related reporting is low, with female researchers being more likely than male researchers to report sex/gender-based differences, which are often published in lower impact journals [8]. This is further evidence of gender bias within research processes and demonstrates the lack of value placed on research focusing on women’s health or comparisons between or among males/men and females/women.

When considering treatments for diseases, to date, more attention has been paid to male-female differences in the efficacy, adverse reactions, and compliance with traditional chemically synthesized drugs [9], with less consideration given to sex and gender differences in the adverse reactions, efficacy, and uptake of biologics, including those that impact the immune system (i.e., immunotherapies). Biologics are a category of drugs that are derived from natural sources. Rather than being chemically synthesized like drugs, biologics are complex substances that are manufactured through synthetic biology methods and have revolutionized treatments for a variety of diseases, including autoimmune diseases, infectious diseases, and cancers. Our goal is to provide empirical evidence that human females and males differ in the outcomes following use of immunotherapies for the treatment of autoimmune diseases (e.g., rheumatoid arthritis [RA]), infectious diseases (e.g., influenza), and solid tumor cancers. In addition, we aim to illustrate commonalities across biologics in which females/women experience/report more adverse reactions than males/men and explain why outcomes are better for females/women than males/men when stimulating rather than repressing immunity with biologic therapies (Fig. 2). Lastly, we suggest avenues for future studies that address the biological as well as sociocultural causes of sex and gender disparities in the effectiveness of biologic therapies in clinical settings.

**Fig. 2:**
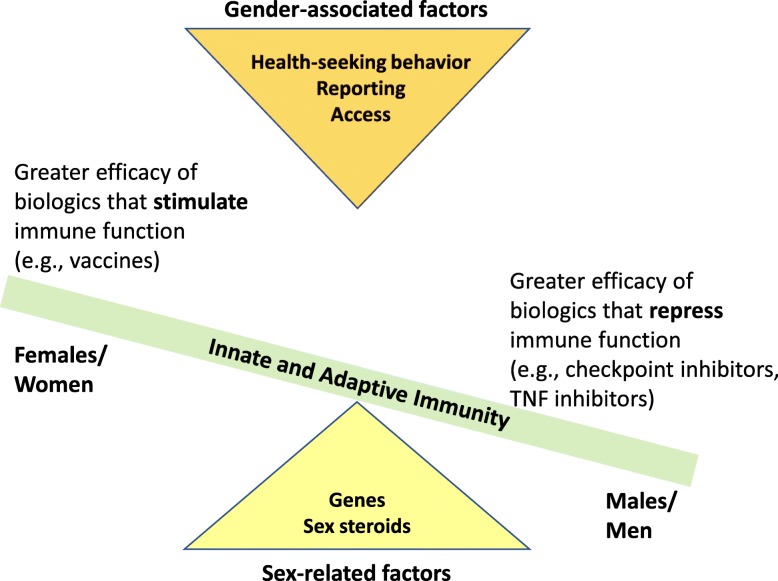
Hypothesized sex- and gender-based factors contributing to differences between females/women and males/men in the efficacy of immunotherapies. Based on the available data, we hypothesize that immunotherapies that stimulate the immune responses are more efficacious in females/women, whereas treatments that repress immune responses are more efficacious in males/men. Biologically, females generally have greater immune responses than males. We hypothesize that sex differences in immune function are caused by sex chromosomal (genetic) and sex steroidal (hormonal) differences between the sexes that differentially affect immune responses to immunotherapies. Sociocultural factors, including health-seeking behaviors, access to healthcare, and reporting of adverse events, also contribute to differences between women and men in immunotherapy adherence and reporting of adverse events. Together, both sex and gender contribute to differential efficacy of immunotherapies

## Male-female disparities in immunotherapies

### Anti-TNF treatment for rheumatoid arthritis (RA)

Rheumatoid arthritis is an autoimmune disease in which the immune system attacks joint antigen causing inflammation and damage to the joints. Rheumatoid arthritis is more prevalent among females than males (3:1 ratio), with females often having more severe disease with onset at a younger age than males [10]. The first-line therapies, which include steroidal and nonsteroidal anti-inflammatory drugs and disease-modifying anti-rheumatic drugs, help manage inflammation, pain, and swelling, but biologics, including tumor necrosis factor (TNF) inhibitors (e.g., etanercept, infliximab, and adalimumab), can result in remission, not only of RA but of other inflammatory diseases, including psoriatic arthritis and inflammatory bowel diseases [11]. While diverse biologics are available for the treatment of RA (e.g., anti-CD20 antibody, rituximab or Janus kinase inhibitor, tofacitinib) and psoriatic arthritis, TNF inhibitors have been most widely used, have been on the market for a long time, and are the only body of data pertaining to male-female disparities in treatment outcomes. Although TNF inhibitors are used for other autoimmune diseases, including systemic lupus erythematosus (SLE) [12] and Sjögren’s syndrome [13], data have not been disaggregated and analyzed to determine if there are male-female differences in treatment responses.

Remission rates are highest when TNF inhibitors are administered in early as opposed to during established RA [14]. During early RA, several cohort studies, in diverse countries in Europe and North America, reveal that remission rates are lower for females than males who are taking TNF inhibitor therapies [15–18]. In fact, male sex is an independent predictor of sustained clinical remission in early RA patients on TNF inhibitors [14]. In contrast, female sex is associated with increased risk of TNF inhibitor therapy failure in Sweden [19]. In a Canadian cohort study, during TNF inhibitor therapy, female RA patients reported more fatigue, worse function, and had higher disease scores than males [20]. In Germany, males are prescribed biologic therapies more often than females [21]. Adherence to biologic therapies is also greater for males than females, with adverse reactions and ineffective treatment being the most common reasons for termination of therapy [22]. In a meta-analysis of almost 100 studies from diverse countries, female sex was an independent risk factor associated with discontinuation of biologic therapies for RA [23].

Consistent with findings from RA, some forms of spondyloarthritis (e.g., psoriatic arthritis), which are classified as autoinflammatory rather than autoimmune diseases, are more prevalent in females than males, with females having different disease manifestations (e.g., inflammatory bowel disease) that can delay diagnosis [24]. Data from observational cohort studies suggest that TNF inhibitors for psoriatic arthritis are more effective in male than female patients, even when controlling for confounding variables, including comorbidities (e.g., chronic pulmonary disease) and lifestyle (e.g., smoking) [25]. Females also are less likely than males to adhere to treatment with TNF inhibitors for spondyloarthritis, with lack of efficacy and adverse events being associated with immunotherapy withdrawal among females [24, 26].

Although numerous studies, including in animals, have addressed the causes of sex disparities in autoimmune diseases, including RA [27], to date, no studies have addressed the mechanisms mediating how immunotherapies, including TNF inhibitors, have greater efficacy and fewer reported adverse reactions in males compared with females. Furthermore, to date, no studies have evaluated reporting of adverse reactions or adherence to immunological biologics for treatment of autoimmune diseases based on the sociocultural construct of gender. Thus, considerable research in this area is required.

### Seasonal and pandemic vaccines for influenza

Vaccines are the best prophylactic treatment for infectious diseases as they provide immunity and protection prior to infection. Sex and gender impact vaccine acceptance, responses, and outcomes [28]. Females are often less likely to accept vaccines [29], but once vaccinated, develop higher antibody responses (i.e., primary correlate of protection) and report more adverse reactions to vaccines than males [28]. For example, after vaccination against influenza, yellow fever, rubella, measles, mumps, hepatitis A and B, herpes simplex 2, rabies, smallpox, and dengue viruses, protective antibody responses are twice as high in adult females as compared with males [28]. Because seasonal influenza vaccines are offered annually, these vaccines provide a larger body of literature than other vaccines, upon which to evaluate sex and gender-based differences.

The acceptance of seasonal influenza vaccines differs between males and females, with vaccine hesitancy reported as being higher among females than males [29, 30], and receipt of seasonal influenza vaccines being greater among males than females [31–33]. Although sex can lead to differences in biological response to vaccines, it does not explain why more females than males are reluctant to be vaccinated, or why in some instances more males than females receive an influenza vaccination. Greater acceptance and receipt of influenza vaccines among males is reported among both older and younger adults. One hypothesis for female-biased reluctance to receive the influenza vaccine is that pregnant women are more likely to have concerns about vaccine safety than the general population due to concerns for their unborn child. Studies suggest, however, that pregnancy cannot be the only explanation of these gender differences [30]. A gender analysis of vaccination acceptance is needed to help explain these differences.

Following receipt, sex differences in immune responses to influenza vaccines occur to a greater extent in adult than aged individuals (65+ years of age) [34]. Data from human trials have shown that when young adults, ages 18–49 years, are administered either a full or half dose of the seasonal trivalent inactivated influenza vaccine (TIV), females generate hemagglutination inhibition (HAI) antibody titers that are twice as high as those of males [35]. Similarly, adult females 20–89 years of age (not partitioned by age or reproductive status (i.e., pre- versus post-menopause)) generate greater neutralizing antibody titers following seasonal TIV than males [36]. In response to the pandemic monovalent 2009 H1N1 vaccine, adult (18–45 years of age) females develop greater IL-6 and antibody responses than adult males, with diminished differences between the sexes among aged individuals (65+ years of age) [37]. Reduced male-female differences in immune responses to the monovalent 2009 H1N1 vaccine among aged individuals are partly due to reproductive senescence in females, in which higher circulating estradiol concentrations in females are associated with greater antibody responses to the vaccine [37]. More trials, including with vaccines other than influenza vaccines, must include sex and gender reporting and analyses to confirm the universality of female-biased immunity following vaccination, at least among adults of reproductive ages.

Passive reporting of local reactions (e.g., muscle pain, redness, and inflammation) to influenza vaccines is consistently more frequent for females than males among both younger and older adults [38]. Measurements of local erythema and induration, both of which are associated with inflammation, reveal that both younger and older adult females have larger (> 6 mm) injection site reactions to TIV than their male counterparts [39]. Systemic reactions (e.g., fever, chills, nausea, headaches, and body aches) to TIV are also more commonly reported by females than males, with fatigue and headache being the most notable systemic reactions that occur more frequently in adult females than males [40]. Reports of local and systemic adverse reactions are also more frequent among adult females than males following receipt of the inactivated monovalent 2009 H1N1 vaccine [41, 42]. To date, whether altering the dose, route for administration of the vaccine, or frequency of administration, could reduce adverse reactions in females that has not been reported. Some of these differences may be due to gender differences in reporting, with men being less likely to report adverse reactions due to a perceived need to appear healthy and strong. Studies have shown that men and women report pain differently, with men often being less willing to report pain [43].

### Checkpoint inhibitors for cancer

Activation of the immune system is integral for fighting cancer. Cytotoxic T lymphocytes (CD8+ T cells) can be activated by tumor antigens to effectively kill cancerous cells as long as their responses are not constrained by negative regulators (e.g., cytotoxic T lymphocyte-associated protein 4 [CTLA-4] and programmed death [PD]-1) that serve as checkpoints to control immune reactions and either limit or prevent tissue damage caused by an overactive CD8+ T cell response. Checkpoint inhibitors, including monoclonal antibodies against CTLA-4, PD-1, and programmed death-ligand 1 (PD-L1), can result in regression of solid tumors by enhancing T cell immunity, sometimes at the cost of induction of autoimmune-like responses and diseases caused by the loss of T cell tolerance [44]. Survival from cancers, including metastatic melanoma, is significantly improved by administration of checkpoint inhibitors as compared with standard chemotherapy [44].

Sex is an important factor in the pathogenesis and prognosis of many cancers that occur outside of the reproductive tract. For the majority of cancers throughout the life course, the risk of malignancy is higher for males than females [45]. Males have an almost 2-fold greater risk of mortality from all malignant cancers (i.e., excluding sex-specific cancers, such as prostate and breast) than do females [46], with sex-differential outcomes being greatest for larynx, esophagus, bladder, and lung cancers [46]. Gender differences may also contribute to men’s higher risk of mortality from cancers, which include less healthcare seeking behavior and delayed diagnosis. In addition, men’s greater engagement in behavioral risk factors for non-communicable diseases, including cancer, such as smoking, drinking, and unhealthy eating, contributes to their lower overall life expectancy [47].

Even treatments for cancers show sex-specific outcomes, with most trials illustrating better outcomes in males than females. Meta-analyses of phase II and III trials of checkpoint inhibitors reveal that both the overall survival as well as progression-free survival is improved in both males and females that receive checkpoint inhibitors, but to a significantly greater extent in males than females for cancers including melanoma, urothelial, and non-small-cell lung [48–50]. Even meta-analyses of only phase III randomized clinical trials [51] reveal that the beneficial effects of checkpoint inhibitors on overall survival and progression-free survival are greater for males than females, with the male-biased outcomes being more pronounced for anti-CTLA-4 than anti-PD-1/PD-L1 therapies.

In addition to considering the impact of checkpoint inhibitors on immune responses and survival outcomes, consideration is given to toxicity and tolerability, including development of immune-related adverse events. Immune-related adverse events can include dermatologic, endocrine, neurologic, gastrointestinal, respiratory, and musculoskeletal pathologies that can often be limited by steroidal treatments [52]. While ample consideration has been given to the differential constellation of immune-related adverse events that occur based on the type and dose of checkpoint inhibitors, to date, no studies have considered whether these adverse events may occur differently in males than females [9, 53]. Because autoimmune responses occur more frequently in females than males, we hypothesize that the frequency and magnitude of immune-related adverse events, including those that mirror autoimmune-like responses, may be more likely in females than males. Greater consideration should be given to whether the efficacy and toxicity of checkpoint inhibitors differ between the sexes [9, 53]. Because immunotherapies for cancer treatment are a relatively recent therapy option, this could explain the apparent discrepancy between greater death from cancer and greater efficacy of checkpoint inhibitors among males than females. As a result of broader use of checkpoint inhibitors for solid tumor cancers, death rates among males are predicted to decline.

## Mechanistic insights into male-female disparities in immunotherapies

### Sex

Sex differences exist in both innate and adaptive immune responses. The *Toll-like receptor 7* (*TLR7*) gene, encoded on the X chromosome, may escape X inactivation resulting in higher expression levels of *Tlr7* in females when compared to males [54–56]. Exposure of peripheral blood mononuclear cells (PBMCs) to TLR7 ligands in vitro causes higher production of interferon-α (IFNα) in cells from human females than from males [57], and plasmacytoid DCs (pDCs) from female humans and mice have higher basal levels of IFN regulatory factor 5 (IRF5) and IFNα production following TLR7 ligand stimulation [58]. In contrast to TLR7, TLR4 expression is greater on immune cells from males than females, and stimulation with lipopolysaccharide (LPS) results in greater proinflammatory cytokine production by immune cells from males, which can be reversed by removal of androgens in male rodents [59]. PBMCs from human males produce more TNF than PBMCs from females following lipopolysaccharide (LPS) stimulation [60, 61]. Neutrophils from human males express higher levels of TLR4 and produce more TNF than female neutrophils both constitutively and following activation with LPS [62]. Among patients with spondyloarthritis, males have greater circulating concentrations of TNF than females [24], which may be one mechanisms mediating how TNF inhibitors are more effective treatments in males than females with either RA or spondyloarthritis.

With regard to adaptive immune responses, females generally exhibit greater humoral and cell-mediated immune responses to antigenic stimulation, vaccination, and infection than do males [28, 63]. Both basal levels of immunoglobulin [64] and antibody responses are consistently higher in females than males [28, 63, 65]. In humans, global analysis of B cell gene expression signatures reveals that the majority of genes differentially expressed between the sexes that are significantly upregulated in B cells from adult females compared with males [66]. Clinical studies reveal that males have lower CD3^+^ and CD4^+^ cell counts, CD4^+^:CD8^+^ cell ratios, and helper T cell type 1 (Th1) responses than females [67–70]. Females also exhibit higher cytotoxic T cell activity along with upregulated expression of antiviral and proinflammatory genes, many of which have estrogen response elements in their promoters [71].

Both genetic and hormonal mechanisms either alone or in combination contribute to sex-related differences in immunity [72]. Many genes on the X chromosome regulate immune function and play an important role in modulating sex differences in the development of immune-related diseases [73]. For example, as compared with males, females have greater expression and activity of X-linked genes (e.g., *TLR7*) associated with isotype switching in B cells, which is epigenetically regulated to result in greater antibody responses in female systemic lupus erythematosus (SLE) patients [56] and in response to influenza vaccines [55].

Circulating concentrations of sex steroids, specifically testosterone, estrogens, and progesterone, in males and females change over the life course and can directly affect immune function. Receptors for sex steroids have been identified in almost all immune cells and can transcriptionally regulate the activity of both innate and adaptive immune cells [72]. The direct effects of sex steroids on immune function have been reviewed extensively elsewhere [72]. Our focus will be on immune responses relevant to the efficacy of TNF inhibitors, vaccines, and checkpoint inhibitors to provide evidence that these immunological pathways are affected by sex steroid signaling. Production and secretion of cytokines and chemokines, including TNF, are affected sex steroid. For example, in mouse models of RA, ovariectomy (i.e., model of surgery-induced menopause) results in greater joint inflammation, neutrophil migration into joint tissues, and concentrations of TNF, which can be reversed by treatment with either estradiol or estrogen receptor agonists [74]. In men, elevated testosterone concentrations are associated with lower concentrations of diverse inflammatory cytokines, including TNF [75], and may partially contribute to how anti-TNF therapies are more efficacious in males than females.

Relevant to vaccine-induced immunity, in females, estrogens, e.g., 17β-estradiol, induce somatic hypermutation and class switch recombination in B cells via the upregulation of activation-induced deaminase (AID), which contains an estrogen response element [76]. In females, greater concentrations of estradiol are associated with greater influenza vaccine-induced immunity [37]. In contrast, elevated concentrations of testosterone in males, in particular younger aged males (18-45 years of age), are associated with reduced neutralizing antibody responses against influenza vaccine viruses [37]. Thus, the hormonal milieu can have profound effects on the regulation of B cell activity, antibody production, and vaccine efficacy. Estrogens also have been shown to modulate the PD-1/PD-L1 pathway in T cells, contributing to greater autoimmune responses in females as compared with males [77, 78]. Whether sex steroidal effects, including effects of testosterone, on regulatory pathways in T cells contribute to how checkpoint therapies differ in their efficacy in males compared with females and whether these sex-related differences change over the life course requires consideration.

### Gender

The role of gender in biologic therapies for autoimmune diseases, infectious diseases, and cancers continues to remain an under researched area. This may be partly due to gender being socio-culturally constructed and highly context-specific, making it difficult to observe and measure. Gender norms, roles, relations, and resulting inequities, however, can lead to health disparities between men and women [79]. Further, gender is intertwined with sex—to the point that it can be difficult to distinguish one from the other.

There are limited studies exploring gender differences in adverse drug reactions, including biologics. While studies show that adverse drug reactions are more frequently reported in females than males (up to 50–75% more likely in some instances [80]), the explanation of these differences often pertains to sex-related factors, including variations in drug exposure and response due to physiological differences (e.g., body weight and hormones) in the pharmacokinetics and pharmacodynamics of traditional drugs [81]. While many adverse drug reactions are the result of biological responses being different between males and females, reasons that women experience increased adverse reactions, however, may not only be biological, but also social in their etiology. The gender bias which resulted from the underrepresentation of women in clinical trials [4] has had a perverse effect on understanding the biological responses to medications, including adverse reactions, in women compared with men—simply put, the dosages are measured against the male body as opposed to the female one, and drug reactions are often not tested against the female body. In addition, gender norms and attributes have led to disparities in health-seeking behavior and reporting, subsequently leading to delayed cancer or other chronic health diagnoses among men. Despite this recognition, there is little evidence demonstrating these effects. The role of gender in biologic treatment requires increased exploration in research if it is to be adequately addressed.

In explaining differences in adverse reactions between men and women, gender-related factors, including psychosocial, behavioral, or cultural differences, are often recognized as being contributing factors [82]. Norms related to what it means to be a man or a woman, including social roles, health-seeking behavior, and even gender bias in drug prescribing by practitioners, can lead to differences in perception of adverse reactions and subsequent reporting [82]. In an observational study on sex differences in adverse drug reactions, body- or self-image was reported to lead to some adverse reactions being perceived and/or reported more by females compared with males, because the burden was perceived to be higher [82]. For a person to report an adverse reaction, they must see a sign or symptom as problematic or troublesome. Hair loss, for example, is often perceived as more problematic in women than men due to societal expectations of what is attractive.

In addition, women tend to seek health information and care at a greater rate than men [82, 83]. This is not only due to it being more socially acceptable for women to be seen as unwell, but also to the fact that women’s reproductive health needs require them to seek health care at a greater rate than men [84, 85]. As a result, men may be less likely to report an adverse reaction, particularly when it is not perceived as serious. To date, there has been little to no research on gender differences in perceptions and reporting of adverse reactions between men and women, not to mention the ways in which gender intersects with sex to contribute to recorded differences. The intersection of sex and gender becomes even more complex when people of other genders, including transgender individuals, are considered. Transgender men and transgender women, for example, may change their hormonal status through surgery and/or drugs and hormone supplementation, which may lead to different detrimental health outcomes, including risk and incidence of venous thromboembolism, ischemic stroke, and myocardial infarction [86]. Not only are transgender individuals underrepresented in immunotherapy studies but the effect of immunotherapy outcomes on transgender individuals is a grossly under researched area.

## Conclusions

Elevated innate and adaptive immunity in females may be detrimental for the progression of autoimmune diseases, such as SLE and RA, but is beneficial for development of a protective immunity following vaccination or in response to cancers. Evidence shows that men and women respond differently to biologic treatments, with immunotherapies that stimulate immunity generally being more efficacious in females/women than males/men and therapies that repress immunity being more effective in males/men than females/women (Fig. 2). It is important that the sex and gender differences contributing to these disparities are explored and communicated to both medical professionals and the public. Biologic treatments, and the communication around those treatments, must be provided in a gender-sensitive and equitable manner. Women need to know that they might react differently to treatment, and that it may take longer to get the dosage correct, as lack of this knowledge may lead to reduced compliance [87] or even vaccine hesitancy [34]. In addition, both men and women need to be encouraged to seek health information and care and report adverse reactions, regardless of how seriously perceived, to medical professionals. Without a sex- and gender-sensitive and equitable approach to biologic treatments, disparities in outcomes will persist and may affect clinical treatment of autoimmune diseases, infectious diseases, and cancers.

## Data Availability

Not applicable

## Abbreviations

CTLA-4: Cytotoxic T lymphocyte-associated protein 4
FDA: Food and Drug Administration
GAO: Government Accountability Office
Th1: Helper T cell type 1
HAI: Hemagglutination inhibition
IFNα: Interferon-α
LPS: Lipopolysaccharide
NIH: National Institutes of Health
PBMCs: Peripheral blood mononuclear cells
pDCs: Plasmacytoid DCs
PD-1: Programmed death
PD-L1: Programmed death-ligand 1
RA: Rheumatoid arthritis
SLE: Systemic lupus erythematosus
TLR4: Toll-like receptor 4
TLR7: Toll-like receptor 7
TIV: Trivalent inactivated influenza vaccine
TNF: Tumor necrosis factor
